# The Evolving Role of Coliforms As Indicators of Unhygienic Processing Conditions in Dairy Foods

**DOI:** 10.3389/fmicb.2016.01549

**Published:** 2016-09-30

**Authors:** Nicole H. Martin, Aljoša Trmčić, Tsung-Han Hsieh, Kathryn J. Boor, Martin Wiedmann

**Affiliations:** Milk Quality Improvement Program, Department of Food Science, Cornell UniversityIthaca, NY, USA

**Keywords:** coliform, dairy foods, indicator organisms, *Pseudomonas*, pathogens

## Abstract

Testing for coliforms has a long history in the dairy industry and has helped to identify raw milk and dairy products that may have been exposed to unsanitary conditions. Coliform standards are included in a number of regulatory documents (e.g., the U.S. Food and Drug Administration’s Grade “A” Pasteurized Milk Ordinance). As a consequence, detection above a threshold of members of this method-defined, but diverse, group of bacteria can result in a wide range of regulatory outcomes. Coliforms are defined as aerobic or facultatively anaerobic, Gram negative, non-sporeforming rods capable of fermenting lactose to produce gas and acid within 48 h at 32–35°C; 19 genera currently include at least some strains that represent coliforms. Most bacterial genera that comprise the coliform group (e.g., *Escherichia*, *Klebsiella*, and *Serratia*) are within the family Enterobacteriaceae, while at least one genus with strains recognized as coliforms, *Aeromonas*, is in the family Aeromonadaceae. The presence of coliforms has long been thought to indicate fecal contamination, however, recent discoveries regarding this diverse group of bacteria indicates that only a fraction are fecal in origin, while the majority are environmental contaminants. In the US dairy industry in particular, testing for coliforms as indicators of unsanitary conditions and post-processing contamination is widespread. While coliforms are easily and rapidly detected, and are not found in pasteurized dairy products that have not been exposed to post-processing contamination, advances in knowledge of bacterial populations most commonly associated with post-processing contamination in dairy foods has led to questions regarding the utility of coliforms as indicators of unsanitary conditions for dairy products. For example, *Pseudomonas* spp. frequently contaminate dairy products after pasteurization, yet they are not detected by coliform tests. This review will address the role that coliforms play in raw and finished dairy products, their sources and the future of this diverse group as indicator organisms in dairy products.

## Introduction

In microbiological testing, an “indicator organism” is defined as a marker that reflects the general microbiological condition of a food or environment (Chapin et al., 2014). In contrast, an “index organism” is a marker that reflects the possible presence of ecologically similar pathogens, suggesting a potential public health risk (Chapin et al., 2014). For nearly a century, coliforms have been used as indicator organisms, first in evaluating water for fecal contamination and later in identifying unsanitary conditions in pasteurized dairy products and other foods. Indeed, coliform testing of pasteurized milk was recommended by the U.S. Public Health Service in the earliest edition of the Grade “A” Pasteurized Milk Ordinance (PMO) published in 1924 (Tortorello, 2003). Currently, the PMO limits coliforms in Grade “A” pasteurized milk and milk products to 10 or fewer CFU per mL (FDA, 2015). Coliforms, defined as aerobic or facultatively anaerobic, Gram-negative, non-spore-forming rods capable of fermenting lactose with the production of acid and gas at 32–35°C (Davidson et al., 2004), were originally considered to represent only strains from the genera *Citrobacter*, *Enterobacter*, *Escherichia*, and *Klebsiella*. Classification of coliforms has been a difficult issue for decades. Coliform differentiation was originally primarily based on the fermentation of sucrose and dulcitol, production of indole and acetylmethylcarbinol, and gelatin liquefaction. Later, Parr established the IMViC formula, which involved indole production, methyl red reaction, Voges-Proskauer test, and citrate utilization (Parr, 1938). Even with these methodological improvements, some strains were still not detected as part of the coliform group.

As taxonomic classification methodologies have improved over the decades, it has become clear that coliforms, as defined solely by the method used to detect them, are a much broader and more diverse group of bacteria (Leclerc et al., 2001). Currently, 19 genera have member strains that fall into the coliform group, mostly encompassed in the family Enterobacteriaceae, however, strains of *Aeromonas*, in the family Aeromonadaceae, also have been identified as coliforms (Abbott et al., 2003) because of their ability to ferment lactose to form gas and acid within 48 h at 32–37°C, although it should be noted that there is some disagreement regarding whether *Aeromonas* should be considered a coliform. Importantly, because of the method-defined nature of this group, it is not uncommon for some species or strains within a genus to be coliform-positive while others are coliform-negative. Such variability within genera complicates classification and understanding of these microorganisms.

In an effort to increase functional differentiation within the diverse coliform group, Leclerc et al. (2001) proposed three categories of coliforms based on taxonomic and physiological traits: “thermophilic,” which include *Escherichia coli* of fecal origin; “thermophilic and ubiquitous” and; “psychrotrophic,” which are purely environmental. Of the “thermophilic” coliforms, which are characterized by their ability to grow and ferment lactose at 44–45°C, the only reliable indicator of fecal contamination is *E. coli*. This organism does not survive well in environments outside of the intestinal tract of warm-blooded animals, hence, it is not an environmental contaminant. However, while others in this group, including some species of *Klebsiella*, *Enterobacter*, and *Citrobacter*, may originate from fecal matter, they also can originate from environmental sources, making them unreliable indicators of fecal contamination. In contrast, “psychrotrophic” environmental coliforms have the ability to grow and ferment lactose at refrigeration temperatures, but generally do not grow above 38°C, which distinguishes them from the thermophilic group. Members of the genera *Klebsiella*, *Enterobacter*, *Serratia*, and others are considered environmental coliforms (Leclerc et al., 2001). Finally, “thermophilic and ubiquitous” coliforms originate from various natural environments including soil, water, vegetation, insects, farm produce, wooden reservoirs, grass, silages, and fresh vegetables (Seidler et al., 1975). Members of this group of “ubiquitous” coliforms are found within the genera *Klebsiella*, *Enterobacter*, and *Citrobacter*.

As a consequence of the improved understanding of the environmental sources of many microorganisms that test positive as coliforms, many industries have moved away from using detection of total generic coliforms for food and water testing (Leclerc et al., 2001; Busta et al., 2006) as they are poor indicators of fecal contamination and overall hygienic conditions. However, coliform testing remains a cornerstone of microbial testing in the U.S. dairy industry, from raw milk testing to processed dairy product testing. Recent studies provide evidence that coliform testing should be reconsidered as a marker for unsanitary conditions in the dairy industry as further understanding of this diverse group of microbes is achieved.

### Coliforms Represent a Common Raw Milk Contaminant that Originates from Various Environmental and Fecal Sources

Coliforms are among the many groups of microorganisms that are normally present in raw milk, i.e., 96% of all bulk tank milk samples collected during a 2002 study in the U.S. were coliform-positive (Van Kessel et al., 2004). California has established the only regulatory limit for coliforms in raw milk intended for Grade “A” dairy products in the U.S. (not to exceed 750 CFU/mL; California Department of Food and Agriculture [CDFA], 2016). Reported coliform levels in raw milk vary greatly, with mean coliform counts for milk sampled in the U.S. ranging from 31 cfu/mL (Boor et al., 1998) to 2,570 cfu/mL (Jayarao and Wang, 1999). Similar results have been reported by others (D’Amico et al., 2008; Pantoja et al., 2011; Jackson et al., 2012). Common coliform genera in raw milk include *Citrobacter*, *Enterobacter*, *Escherichia*, and *Klebsiella* (Jayarao and Wang, 1999), which can originate from a variety of sources in the dairy farm environment including water, plant materials, equipment, dirt, and fecal sources (Kagkli et al., 2007). High levels of coliforms (e.g., >1,000 cfu/mL) in raw milk may indicate unsanitary practices on the farm, inadequate refrigeration, or the presence of coliform mastitis (Jayarao and Wang, 1999; Hogan and Smith, 2003, Pantoja et al., 2011). Additionally, certain management practices at the farm level, including milking machine wash failures, rate of cluster washes and rate of milking unit fall-off during milking also correlate to variations in levels of coliforms in raw milk (Pantoja et al., 2011).

Milking mastitic cows can introduce coliforms into bulk tank raw milk, hence somatic cell counts (SCC) also can be correlated with the presence of coliform bacteria. Coliform genera recognized as causing mammary infections include *Escherichia*, *Klebsiella*, *Enterobacter*, and *Serratia* (Hogan and Smith, 2003). The cow may become exposed to mastitis pathogens through manure, bedding, soil, and water (Hogan and Smith, 2003). Pantoja et al. (2011) found that in-line coliform counts increased 6.3% for every 10% increase in in-line SCC, which could reflect as little as the milk from one mastitic cow being milked into the bulk tank.

Despite there being no federal coliform regulation for raw milk being processed into U.S. Grade “A” dairy products, many states that allow the sale of raw milk for direct human consumption have regulatory limits for coliforms. For example, in California, raw milk “shall contain not more than 15,000 bacteria per milliliter or [not] more than 10 coliform bacteria per milliliter” (California Food and Agriculture Code, 2016). According to a Raw Milk Survey conducted by the National Association of State Departments of Agriculture (NASDA; Ehart) in 2011, 30 states allowed raw milk sales. Five of the thirty states had special regulations for raw milk, including “cow-share” agreements, in which the consumer “owns” all or part of a cow, and therefore, can have access to its milk, or limit raw milk sale to specific markets. Among the 30 states, twelve allow the consumer to access milk at both the farm where the milk is produced and at retail stores that can be separate from the farm. The remaining thirteen states restrict legal sales of raw milk only to the farm where the milk is produced. Of the 30 states that allow sale of raw milk for human consumption, coliform limits of ≤10 cfu/mL to ≤100 cfu/mL are imposed in 20 states (**Table 1**; Ehart, 2011).

**Table 1:** Summary of coliform standards for raw milk sold for human consumption.

	Number of states allowing raw milk sales^1^		
Coliform standard^2^	On-farm sale	Retail milk sale	Cow-share/Other^3^
No limit	8	0	2
≤10 cfu/mL	4	9	2
≤25 cfu/mL	0	1	0
≤50 cfu/mL	0	2	0
≤100 cfu/mL	1	0	1
Total	13	12	5

*^1^For details on raw milk regulations by state, see Ehart (2011); as state-level raw milk regulations change frequently, states are not listed here to avoid mis-leading or out-of-date information. ^2^cfu = colony forming units. ^3^A cow-share is an agreement entered into by individual(s), who pay a farmer a fee for boarding and milking the cow(s) that they own. After the cows are milked, the individual(s) obtain the milk from the farmer. Technically, these arrangements are not considered “raw milk sales.”*

While the use of coliforms as indicator organisms for the presence of unsanitary conditions in milk handling is increasingly under scrutiny, it is clear that coliforms are not appropriate index organisms for the presence of public health hazards in dairy products. For example, Jackson et al. (2012) examined levels of coliform bacteria in raw silo milk in correlation to the presence and levels of four pathogens of interest (*Bacillus cereus*, *E. coli* O157:H7, *Listeria monocytogenes*, and *Salmonella* spp.). The study concluded that there were no significant increases in coliform levels in pathogen-positive samples as opposed to pathogen-negative samples. Similarly, no significant differences existed in coliform counts from samples with zero, one, two, three, or four pathogens detected. These results illustrate that coliform counts are not an index of the presence of these four pathogens, and that coliform testing of raw milk intended for human consumption cannot be used to reliably identify raw milk that presents a public health risk. This is also consistent with other studies (D’Amico et al., 2008) that detected pathogens in raw milk samples that had very high microbiological quality and low coliform counts.

### Coliform Contamination in Pasteurized Fluid Milk Leads to High Total Bacteria Counts and Low Sensory Scores

Coliform testing has been used to indicate hygienic condition of dairy products for nearly a century. Coliforms are common contaminants in fluid milk (Carey et al., 2005; Martin et al., 2012), cheeses (Wolfe et al., 2014; Trmčić et al., 2016) and other dairy products. Recent studies have shown post-processing contamination (PPC) with coliforms in 7.6–26.6% of U.S. fluid milk samples tested between 2001 and 2010 (Martin et al., 2012). Pasteurized fluid milk samples that were contaminated with coliforms had significantly higher bacterial counts and significantly lower overall sensory scores (Martin et al., 2012) over shelf-life than samples that tested negative for coliforms. The PMO limits the number of coliforms in pasteurized grade “A” milk to no more than 10 cfu/mL throughout shelf-life (FDA, 2015). In general, due to the heat labile nature of these organisms, the presence of coliforms and other Gram-negative bacteria in pasteurized fluid milk indicates: (i) PPC of the product; or (ii) pasteurization failure. Many coliforms in pasteurized fluid milk products are psychrotolerant, and thus able to grow to high levels at refrigeration temperatures (Carey et al., 2005; Ranieri and Boor, 2009; Martin et al., 2011; Masiello et al., 2016).

A recent study of coliform bacteria in pasteurized fluid milk indicated that species of *Enterobacter*, *Hafnia*, *Citrobacter*, *Serratia*, and *Raoultella* represented the majority of the coliform population (Masiello et al., 2016). Of the coliform isolates collected by Masiello et al. (2016), the majority showed the ability to grow substantially (i.e., >5 log growth) over 10 days at refrigeration temperatures. This robust growth, accompanied by the ability of many psychrotolerant coliforms to produce lipolytic and proteolytic enzymes (Wessels et al., 1989; Nornberg et al., 2009; Masiello et al., 2016) which are capable of causing flavor, odor and body defects in fluid milk, make the presence of coliforms in fluid milk detrimental to quality and consumer acceptance.

Prevention of PPC with coliforms and other microorganisms remains a major hurdle for some dairy processors in the U.S. (Ralyea et al., 1998; Ranieri et al., 2009; Martin et al., 2011). In many cases, contamination can be traced back to the presence of biofilms in processing equipment. Many types of bacteria are capable of forming biofilms in equipment, especially in cracks, dead ends and gaskets. Biofilms, which have been described as a functional consortium of microorganisms attached to a surface and embedded in the extracellular polymeric substances produced by the microorganisms (Costerton et al., 1987), allows colonization of populations of microorganisms and provides protection for the microbes from cleaning and sanitization procedures. As the biofilm matures, cells slough off and can contaminate product during processing (Kumar and Anand, 1998). In dairy processing, in particular, the use of clean-in-place (CIP) systems may unintentionally lead to biofilm formation because such systems may fail to remove accumulated microorganisms and organic materials effectively (Kumar and Anand, 1998). The formation of the biofilm begins with a process known as conditioning which begins 5–10 s after milk processing begins (Marchand et al., 2012). In particular in processes where temperatures are high enough to begin to denature whey proteins (i.e., 65°C), adherence of this layer to the surface alters the surface properties and improves the ability of bacterial contaminants to adhere (de Jong, 1997). Continuation of the process of biofilm formation, namely bacterial adhesion, bacterial growth and biofilm expansion (Marchand et al., 2012) leads to biofilms that are resistant to removal, especially using CIP systems. Stringent cleaning and sanitation practices along with attention to sufficient preventative maintenance, hygienic design and employee training are essential to minimize formation of biofilms and prevent PPC.

### Coliforms in Cheese Represent a Diverse Group of Organisms

Coliforms are widely found in many cheeses (Khayat et al., 1988; Brooks et al., 2012). However, in contrast to the presence of these microbes in raw and pasteurized fluid milk, and even in some other cultured products (e.g., yogurt), the presence of coliforms in cheese may not necessarily be negative. The vast variety of types of cheese manufactured contributes to the complexity of fully understanding the role of coliforms in cheese quality and safety. Cheese product characteristics, including moisture content, pH, salt content, ripening conditions, age of product, and culture all influence potential levels of and roles for coliforms and other microorganisms in the final product (Wolfe et al., 2014; Trmčić et al., 2016). A survey of raw milk cheeses by Brooks et al. (2012) found that 5 of 41 commercially available raw milk cheese samples had detectable coliforms (i.e., >10 cfu/g). In a similar study, Trmčić et al. (2016) surveyed 273 raw and pasteurized cheeses from the U.S. and other countries and found that 75 of those samples were positive for coliforms in concentrations above 10 cfu/g.

Many individual states in the U.S. have limits of 10 or 100 cfu/g for coliforms in cheese. In the European Union (EU), where microbiological specifications are regulated by the European Commission (EC), there are no regulations concerning coliforms (EC No 2073/2005) for cheese products. Regulations, instead, are focused on *Salmonella*, coagulase positive *Staphylococci* and *E. coli*. Additionally, regulations set forth by the EC are categorized by type of product (i.e., cheese made from raw milk or from thermized milk, soft cheese, fresh cheese and other cheeses), thus acknowledging the need for a scientific approach to assessing the hygienic conditions and microbial food safety hazards associated with cheeses.

Sources of coliforms in cheese products can vary depending on the product. Due to the nature of raw milk cheeses, the presence of coliforms is not unexpected as coliforms are common in raw milk. However, in pasteurized cheese products, coliforms present in raw milk should have been eliminated by pasteurization, implying that any coliforms present in the finished product resulted from PPC. Recontamination can occur in the processing or aging facility through cheese contact with contaminated water, humans, air, and biofilms on equipment (Lawrence and Lilly, 1972; Dancer et al., 1997; Hughes, 2003; Kilb et al., 2003).

High levels of coliforms in pre-cultured milk intended for cheese making may have deleterious effects on cheese production, specifically if acid development by the lactic acid bacteria (LAB) occurs more slowly than desired. Growth of coliforms early in cheese production may lead to early blowing, or gas production defects in the product (Farkye, 2000; Ledenbach and Marshall, 2009). Additional effects and byproducts of coliform growth early in cheese production can be reduction of desirable formation of diacetyl (Ledenbach and Marshall, 2009), lactic acid, acetic acid, formic acid, succinic acid, ethanol, and 2,3-butyleneglycol (Farkye, 2000).

The growth or death of coliforms in cheese products depends on a variety of parameters including cheese pH, age, moisture content, salt content, free fatty acid content and others. Nunez et al. (1985) found that Manchengo cheese products made with cooked curd had higher levels of coliforms than those made with uncooked curd. This difference was attributed to lower pH in the uncooked curd (due to superior growth of LAB). Nunez et al. (1985) also found that the temperature of ripening had a significant effect on the reduction of coliforms, concluding that an aging temperature of 15°C was the optimum temperature to achieve reduction in coliforms (and other unwanted bacteria) and also to protect desired sensory attributes. Coliforms are typically inactivated and/or inhibited by the drop in pH during cheesemaking acidification. If pH increases during aging (due to proteolysis, typically in surface ripened cheese), however, conditions may exist to support coliform growth (Ledenbach and Marshall, 2009). Finally, Trmčić et al. (2016) reported that pasteurization, pH, water activity, milk type (e.g., cow milk), and rind type were cheese factors that significantly influenced detection of coliforms in cheese. They also report that water activity is significantly associated with the final concentration of coliforms in cheese; suggesting more than 0.5 log cfu/g higher average final concentration of coliforms for every 0.01-unit increase in water activity.

Proteolytic and lipolytic enzyme production varies greatly in the coliform group (Wessels et al., 1989). Enzyme production is largely dependent on product storage temperature. Proteolytic and lipolytic enzymes can contribute both desirably and undesirably to flavor and texture characteristics of cheese. The proteolytic activities of some strains of coliforms have been studied (Macedo and Malcata, 1997; Nornberg et al., 2009); some are highly proteolytic. To date, studies have primarily focused on the negative impact of enzymatic activity from coliform origin on dairy product quality, but some work has examined possible advantageous impacts that coliform enzymatic activity may have on ripening and flavor development of certain cheeses (Macedo and Malcata, 1997). The notion of coliforms as possible desirable contributors to the complex ecosystem of cheeses, particularly farmstead and artisan cheeses, is supported by studies suggesting that coliforms may be part of the natural microflora of at least some cheeses (Quigley et al., 2011). Further, as different Gram-negative bacteria are being identified as having a high potential for production of aroma compounds during cheese production, new bacterial cultures are being developed to utilize this potential. Some of the species used in these new bacterial cultures (e.g., *Hafnia alvei*) are members of coliforms/Enterobactriaceae in which case the use of these bacterial groups as indicators would not be appropriate (Morales et al., 2003; Deetae et al., 2009).

In the U.S., testing dairy products for coliforms (beyond fluid milk and cheese) is required by the PMO. Coliform limits in cultured products (e.g., yogurt), ice cream, non-fat dry milk and others are set at ≤10 cfu/ml or g (FDA, 2015). Current standard methods recommend testing yogurt for coliforms within 24 h of production to obtain meaningful results (Duncan et al., 2004). However, enumerating *Enterococcus* may provide a more reliable hygiene indicator than coliforms because they are more likely to survive in the low pH environment (Frank and Yousef, 2004). There is little research on the use of Enterococci as indicators in high acid dairy products, however, Birollo et al. (2001) concluded that Enterococci have little industrial use as hygiene indicators in yogurt processing. While the pH of yogurt has long been considered too low to allow survival of coliforms, limited evidence exists to support this conventional wisdom. A recent study by Hervert (2016) evaluated a variety of common coliforms, *Enterobacteriaceae* (EB) and non-EB Gram-negatives (e.g., *Pseudomonas*) for their abilities to survive in commercial yogurt products. The study showed that, in general, coliform and EB organisms were capable of surviving and, sometimes, even growing under conditions encountered in commercial yogurt products, while non-EB Gram-negative bacteria showed rapid die-off. The authors concluded that testing for EB provided the most comprehensive approach for monitoring hygiene indicators in yogurt as opposed to testing for coliform and total Gram-negative bacteria.

Coliform contamination in ice cream has not been widely or recently studied in the U.S., although surveys from other countries indicate that coliform levels range from less than detectable to >10^4^ cfu/g (Massa et al., 1989; Warke et al., 2000; M-E-Elahi et al., 2002; El-Sharef et al., 2006). The storage conditions of ice cream are generally thought to inhibit growth of bacterial contaminants, including coliforms. As a heat-treated product, the presence of coliforms in ice cream and other frozen dairy products is an indicator of PPC. However, because contaminated ingredients (e.g., nuts, fruits, etc) may be added to the product after pasteurization, there is considerable opportunity for bacterial contamination that does not originate from unhygienic conditions, *per se*, in the processing facility (Duncan et al., 2004).

### A Century of Coliform Testing – Time to Rethink Our Indicator Organisms in the Dairy Industry?

As the landscape of the global and U.S. food industries changes and responds to new requirements to ensure a safe food supply, there is reason to review traditional methods of evaluating dairy product hygiene and safety. Because of their heat-labile nature, coliforms long have been used in the dairy industry as indicators of PPC. Certainly, in general, coliforms are undesirable in processed dairy products (e.g., fluid milk). However, while coliforms do represent PPC and can cause flavor, odor and body defects in many dairy products, in some dairy products, detection of this group of microbes is insufficient for identifying unhygienic conditions.

Recent work indicates that testing for EB or total Gram-negative bacteria offers a distinct advantage to coliform testing when detecting common PPC organisms in dairy products (Hervert et al., 2016). EB is a taxonomic group of microorganisms that encompasses almost all of the coliform group (Hervert et al., 2016) with the exception of *Aeromonas*, and has been used as a hygiene indicator broadly in Europe (European Communities Regulation, 2010). A benefit of testing for EB over coliforms is increased sensitivity for detecting PPC because of the broader range of contaminants detected (Hervert et al., 2016). Although the EB group includes some pathogenic bacteria (e.g., *Salmonella*), EB are considered indicators as opposed to index organisms. In general, their presence in some food products has no correlation with the presence of pathogens (Johnson, 1996), although this has not been studied specifically in dairy foods. Recent work has identified that the EB group is superior as a hygiene indicator in yogurt products because they are capable of surviving, and even growing, under conditions encountered in that product (Hervert, 2016).

Testing for total Gram-negative bacteria as an indicator of unsanitary conditions in certain dairy products (e.g., fluid milk) offers a distinct advantage over coliform or EB testing (**Table 2**). *Pseudomonas*, which lacks the ability to ferment lactose and is therefore not a coliform, has been described as the major contributor to PPC in the U.S. fluid milk industry (Ranieri and Boor, 2009; Martin et al., 2012). *Pseudomonas* readily forms biofilms in processing equipment (Ralyea et al., 1998) and, according to a survey of fluid milk across the U.S., accounts for ~70% of fluid milk spoilage from PPC in the U.S. (Ranieri and Boor, 2009). However, coliform tests do not detect *Pseudomonas* and other non-coliform Gram-negative bacteria that commonly contaminate fluid milk post-processing. Van Tassell et al. (2012) found that crystal violet tetrazolium agar (CVTA) was the most effective selective medium for detecting a diverse group of *Pseudomonas* commonly associated with PPC in fluid milk, whereas commonly employed coliform media (e.g., violet red bile agar) had limited ability to detect *Pseudomonas*. Therefore, coliform testing is not an effective approach for detecting fluid milk exposed to PPC. As dairy plants strive to reduce PPC, the ability to identify contamination occurrences and to rapidly respond is critical to improving the quality of fluid milk products. Based on the current understanding of the ecology of PPC in fluid milk and the inability of coliform testing to identify the majority of these contaminants, exclusive use of coliform testing for this purpose ironically may prevent the fluid milk industry from detecting and rapidly resolving contamination issues.

**Table 2:** Proposed hygiene indicator tests for different dairy products.

Product	Proposed microbial hygiene indicator test^2^	Justification	Key references
Fluid milk	Total Gram-negative bacteria	Key hygienic issues in pasteurized fluid milk are (i) PPC and (ii) pasteurization failure. Both can be detected more reliably with a test that detects all GN bacteria (rather than coliform or Enterobacteriaceae [EB] tests).	Ranieri and Boor, 2009; Martin et al., 2012
Fermented dairy products (e.g., yogurt, kefir, etc)	Enterobacteriaceae (EB)	Non-EB Gram-negative bacteria decline rapidly at the pH encountered in fermented dairy products while EB generally survive in these conditions making it possible to detect them as indicators of unhygienic conditions.	Hervert, 2016; Hervert et al., 2016
Aged cheeses	Targeted risk-based pathogen testing^1^	No suitable tests are currently available, specific pathogen tests are recommended based on risks associated with specific cheese characteristics (e.g., pH, a_w_, etc).	Schvartzman et al., 2014; Trmčić et al., 2016
Fresh cheeses	EB and/or *Escherichia coli* (additional research needed)^2^	Currently coliforms and EB are commonly used as hygienic indicators in fresh cheeses.	
Dairy powders	EB and/or targeted risk-based pathogen testing (additional research needed)^2^	Currently coliforms and EB are commonly used as hygienic indicators, but testing for selected pathogens is typically required for dairy powders that are used in infant formula.	
Ice cream	Total Gram-negative bacteria (additional research needed)^2^	Currently coliforms and EB are commonly used as hygienic indicators in ice cream.	
Butter	Total Gram-negative bacteria (additional research needed)^2^	Currently coliforms, EB, and proteolytic bacteria are commonly used as hygienic indicators.	

^1^*Testing for target pathogens of concern may be appropriate for all products (or required under some jurisdictions), even if not specifically mentioned in this Table.*

^2^*Proposed indicator tests for these four products (fresh cheese, dairy powders, ice cream, butter) are based on product characteristics, processing parameters and research findings from other dairy products; additional research is needed for these specific products to make more definitive recommendations regarding best practices for microbial hygiene indicator tests.*

Further, in the cheese industry, there is growing concern that coliform testing, especially in raw milk cheeses, provides little in the way of indicating hygienic conditions. Some research suggests that certain members of the coliform group, in fact, may be advantageous microorganisms in certain types of cheese (Macedo and Malcata, 1997; Quigley et al., 2011), and that coliforms serve no scientifically valid function as an index organism (i.e., for suggesting pathogen contamination). At best, coliform testing in cheese may provide insight into potential PPC, depending on the product. At worst, coliform testing may provide a false sense of security when public health risks from pathogenic contaminants are present. Trmčić et al. (2016) assessed the association between coliform detection in raw and pasteurized cheeses and the presence of *Salmonella*, *Staphylococcus aureus*, Shiga toxin-producing *E. coli*, *Listeria monocytogenes*, and other *Listeria* species. This study found no association between pathogen presence and coliform detection, despite an association between *Listeria monocytogenes* with washed rind style cheeses. Other groups have also found that cheese characteristics (e.g., pH) are associated with the presence of pathogens in the product (Schvartzman et al., 2014). This is not surprising given the association between cheese characteristics and overall microbial diversity in cheese (Wolfe et al., 2014). The lack of association between the presence of pathogens and coliform detection, as well as the evidence that cheese characteristics are associated with pathogen prevalence, suggests that a model whereby products are categorized by their inherent characteristics and tested for organisms that are likely to cause a public health threat in those particular products provides a more effective approach to assuring public health than coliform testing (**Table 2**).

## Conclusion

Testing for the presence of coliform bacteria, a method-defined group, has long been practiced in the U.S. dairy industry, from raw milk to processed products. Coliform testing is rapid and has long been used as a primary indicator test for hygienic conditions associated with dairy products. However, recent advances in taxonomy and understanding of coliforms has led to questions regarding the suitability of testing for this diverse group of organisms as indicators for unhygienic conditions in dairy products. From fluid milk, where coliforms represent a minor proportion of PPC, to cheese products, where coliforms do not accurately represent public health risks, it is time to rethink the relevance of this century-old indicator group as a means for protecting public health. We propose implementation of appropriate pathogen testing (e.g., *Listeria* testing in washed rind cheeses) or testing for a comprehensive group of all organisms linked to PPC (e.g., total Gram-negative testing in fluid milk) to ensure a high quality and safe dairy food supply.

## Author Contributions

NM, TH, and AT were primarily responsible for literature review. NM, AT, TH, KB, and MW were responsible for preparing the manuscript.

## Conflict of Interest Statement

The authors declare that the research was conducted in the absence of any commercial or financial relationships that could be construed as a potential conflict of interest.
